# Sociodemographic profile and level of burden of dementia patients’ caregivers who participate in a support group

**DOI:** 10.1590/S1980-57642010DN40300012

**Published:** 2010

**Authors:** Lusiêni Diel, Letícia M.K. Forster, Renata Kochhann, Márcia Lorena Fagundes Chaves

**Affiliations:** 1 Developmental Psychology Post-Graduate Course, UFRGS, Psychology Institute, Porto Alegre RS, Brazil.; 2 Dementia Clinic, Neurology Service, Hospital de Clínicas de Porto Alegre, Porto Alegre RS, Brazil.; 3 Medical Sciences Post-Graduate Course, UFRGS School of Medicine, Porto Alegre RS, Brazil.; 4 Internal Medicine Department, UFRGS School of Medicine, Porto Alegre RS, Brazil.

**Keywords:** caregiver, burden, dementia, Alzheimer

## Abstract

The Brazilian Alzheimer’s Association recommend the dementia patient’s caregiver to attend group meetings which aim to give information and enable them to express and share feelings with individuals who are facing similar difficulties.

**Objectives:**

To identify the sociodemographic profile of the individuals who attend the Support Group for Family Members of Individuals with Alzheimer’s disease at Hospital de Clínicas de Porto Alegre, and to verify the degree of burden associated to the care given to this kind of patient.

**Methods:**

Forty-eight participants were sub-divided into two groups: 23 non-caregivers and 25 caregivers. All participants answered a sociodemographic questionnaire, and the caregivers also answered the Zarit Burden Interview (ZBI). Student’s t test was used for comparison of parametric data, and *Chi-square* test for categorical data between caregivers and non-caregivers. Spearman’s *rho* correlation analysis was performed for the ZBI and the studied variables.

**Results:**

Participants were predominantly women. Only age differentiated one subgroup from the other. The mean score on the ZBI was 35.1 (14.7), and most of the caregivers presented up to moderate burden.

**Conclusions:**

Women attended the Support Group either as caregiver or non-caregiver. The level of burden among caregivers of high educational attainment was relatively high besides the short time as caregiver (up to a year).

## Abstract (pt)

A Associação Brasileira de Alzheimer recomenta que os cuidadores de pacientes com demência participem de encontros em grupo que têm por objetivo fornecer informações e permitir que eles expressem e compartilhem seus sentimentos com pessoas que estejam enfrentando as mesmas dificuldades.

**Objetivos:**

Identificar o perfil sociodemográfico dos frequentadores do Grupo de Apoio aos Familiares de Pacientes com Alzheimer do Hospital de Clínicas de Porto Alegre e verificar o grau de sobrecarga resultante deste cuidado.

**Métodos:**

Os 48 participantes que fizeram parte deste estudo foram subdivididos em dois grupos: 23 não cuidadores e 25 cuidadores. Eles responderam a um questionário sociodemográfico sendo que os cuidadores também responderam à Entrevista Zarit de Sobrecarga (ZBI). O teste t de Student foi usado para comparação dos dados paramétricos, e o teste *Qui-quadrado* para dados categóricos entre cuidadores e não cuidadores. A análise de correlação *rho* de Spearman foi feita com a ZBI e as seguintes variáveis quantitativas: idade, filhos, escolaridade, tempo como cuidador e carga horária semanal de cuidado.

**Resultados:**

Os participantes eram na sua maioria mulheres. Apenas a idade diferenciou um subgrupo do outro. O escore médio da ZBI foi de 35,1 (±14,7) e a maioria dos cuidadores apresentou sobrecarga que variou de leve a moderada.

**Conclusões:**

As mulheres participaram do Grupo de Apoio tanto como cuidadoras ou não cuidadoras. O nível de sobrecarga entre os cuidadores de maior nível de escolaridade foi relativamente alto apesar do pouco tempo exercendo tal função (máximo de um ano).

According to the World Health Organization (WHO), by the year 2025, Brazil will be ranked sixth in the world in number of elderly people.^1^ Aging of the population has been observed worldwide and a commensurately high number of individuals, including those with dementia, will need home care generally involving family members.^2^

Alzheimer’s disease (AD) is the most frequent cause of dementia accounting for more than half of dementia cases.^3^ AD is a degenerative pathology which demands progressively greater efforts by the family for the care of patients. Dependence and other consequences caused by the disease exhausts the main caregiver^4^ and on this matter the WHO^5^ advises that one of the goals in attending patients with AD is to promote support for their families. The huge burden imposed on the caregiver is one of the reasons why one should not take on this task alone.^6^

Governmental actions in Brazil fall short in this area of health assistance, which consequently increases the importance of the family caregiver. However, the vast majority of these relatives are uninformed and unassisted.^7^ The Brazilian Alzheimer’s Association recommends family members to attend group meetings which aim to give support, information and enable caregivers to express and share their feelings with people who are facing similar difficulties.^8^

Current studies suggest that this overload can have devastating effects on caregivers. These effects can be categorized as objective (financial problems, social relationships and leisure) and subjective (emotional and physical stress).^6,9^ The most common effects reported are depression, high levels of anxiety and stress, high emotional burden,^9,10^ lower sense of self-efficacy^11^ and impaired immune system.^12^ Studies have proved that the most efficient way to reduce burden in this population of caregivers is to promote group-based psychoeducational interventions.^11^

The most frequently used instruments in studies carried out in Brazil include sociodemographic questionnaires,^7,9^ as well as scales for anxiety, depression^9,13^ and burden.^14-18^ In order to assess burden, the vast majority of studies have applied the Zarit Burden Interview (ZBI).^10,12,19,20^ The ZBI was validated for use in Brazil by Scazufca^21^ in caregivers of mentally ill patients, and after the study of Taub and colleagues^22^ verifying its reliability, this instrument has become widely used in investigations with caregivers of dementia patients. Furthermore, the ZBI has proved to be easy to administer.^12^

The objectives of the present study were to identify the sociodemographic profile of individuals who attended the Support Group for Family Members of Individuals with Alzheimer’s disease, and to rate levels of burden of caregivers who participated in this activity.

## Methods

The present study was carried out in the Support Group for Family Members of Individuals with Alzheimer’s disease at Hospital de Clínicas de Porto Alegre. The meetings occurred on Monday afternoons, as part of a six-month program which was open to the community. This group is coordinated by a neuropsychologist and has the objective to promote health, quality of life, and knowledge about aging and dementia (diagnosis, treatment, management and support). Professionals from different areas are invited to lecture about these topics, such as speech therapists, psychiatrists, neurologists, lawyers, occupational therapists and neuropsychologists.

Caregivers (individuals who spend time taking care of patients with dementia, whether professional or voluntary) and non-caregivers who attended the group at least once in a 3-month period were invited to participate in the study. Only 48 subjects agreed to participate in this cross-sectional study. All participants answered a sociodemographic questionnaire, while only the caregivers answered the Zarit Burden Interview. In this instrument, most of the questions dealt with subjective burden, but a few were related to objective burden, such as financial and social issues. All subjects signed an informed consent before being enrolled in the study. There were no exclusion criteria as the goal was to characterize individuals who attended the group meetings, thus caregivers and non-caregivers were allowed to participate.

The sociodemographic questionnaire was devised by the researchers in order to collect data on gender, age, schooling, occupation and income. It also contained open and closed questions about the caregiver profile, such as time as a caregiver and weekly time of care. The ZBI^19^ consists of a 22-item questionnaire which verifies the burden perceived by the caregivers concerning: request for help from patients, lack of personal time, feeling of tiredness, embarrassment, irritation, tension, lack of privacy, feeling that the caregiver’s social life is impaired, among others. Questions from 1 to 21 are answered through the following levels of frequency: never=0, rarely=1, sometimes=2, quite frequently=3, or nearly always=4. Question 22 assesses, in general terms, the burden felt by the caregiver and can be scored through the following possibilities: not at all=0, a little=1, moderately=2, quite a bit=3, extremely=4. Total scores range from 0 to 88, and the higher the score, the higher the level of burden. According to Karlikaya et al.,^23^ zero to 20 points indicates little or no burden, 21 to 40 points, mild to moderate burden, 41 to 60 points, moderate to severe, and finally 61 to 88, severe burden.

For the purposes of the analysis, the collected data were sub-divided into two groups: caregivers and non-caregivers. Descriptive statistics (mean, SD, and relative frequency) were calculated for demographic data and the ZBI. Student’s t test was used for comparison of parametric data, and *Chi-square* for categorical data between caregivers and non-caregivers. Spearman’s *rho* correlation analysis was done on the ZBI and the following quantitative variables: age, children, schooling, time as a caregiver and weekly time of care. The statistical analysis was carried out with the *Statistical Package for the Social Sciences* for Windows version 18.0. The study was approved by the Ethics Committee for Medical Research of Hospital de Clínicas de Porto Alegre.

## Results

A total of 48 individuals, 23(48%) non-caregivers and 25(52%) caregivers of patients with dementia, participated in the study, all of whom frequented the Support Group for Family Members of Individuals with Alzheimer’s disease at Hospital de Clínicas de Porto Alegre. In both groups, participants were predominantly women (Table 1).

**Table 1:** Characteristics of caregivers and non-caregivers.

Characteristics	Caregiver (N=25)*	Non-caregiver (N=23)*	P value
Gender^+^ (female) - N (%)	18 (72)	18 (78)	0.743
Age^§^ (mean±SD)	54.0±12.0	65.5±12.8	0.003
Schooling^§^ (mean±SD)	11.5±3.5	11.2±3.8	0.765
Marital status^||^ - N (%) Married Single Separated Widow(er)	10 (40) 8 (32) 5 (20) 2 (8)	7 (30) 7 (30) 2 (10) 7 (30)	0.205
Income^+^- N (%) Zero income 1 to 3 minimum wages 4 to 7 minimum wages 8 or more minimum wages	1 (4) 11 (44) 7 (28) 6 (24)	0 (0) 4 (17) 11 (49) 4 (17)	0.185
Working (No)^+^ - N (%)	11 (44)	14 (61)	0.244
Children^§^ (mean±SD)	1.9±1.5 (range: 0-7)	1.4±1.5 (range: 0-5)	0.286
Participation in the support group^+^ - N (%) At least once a month First time or occasionally	11(50)11(50)	5(42)7(58)	0.729

* Some subjects did not answer some of the questions;

+ Chi square test (Fisher's Exact Test);

§ Student's t test;

|| Chi-square test

### Non-caregivers

The non-caregivers were older and their ages ranged from 39 to 80 years old, however almost half of them were ≥70 years. They were widow(er)s (30%), married (30%) or single (30%), and almost half lived alone (43%) and did not have any kinship with a dementia patient (48%).

Nine participants (39%) had graduated in High School, and eight (35%) held a College degree or were undergraduate students. The majority of them (58%) had a monthly income ranging from four to seven minimum wages (1 minimum wage=U$ 353.00). The following occupations were reported: business person, technician, government employee, nurse, nursing assistant, builder, veterinary, dentist and housewife, and frequencies were very low of each category.

When asked about reasons for participating in the support group, the answers were: desire to be able to transmit information about the disease to family, to friends or to volunteer caregivers; intention to understand the dementia patient and help to improve his/her quality of life; curiosity about Alzheimer’s disease; doubt about the symptoms of the disease in order to confirm if they were presenting these symptoms; and seeking information to prevent AD.

### Caregivers

Among the 25 caregivers, two were professional and the others were volunteers. Caregiver age ranged from 24 to 77 years, and more than half were aged ≤60 years. Forty percent were married, and 31% were single. Fifteen participants had High School or College degrees.

The vast majority (76%) of caregivers did not live with the dementia patient. Twenty (80%) participants were related to the dementia patient, with most being their children (12 daughters and two sons). The other caregivers were children-in-law, husband and granddaughter.

Most caregivers had a job (56%) and 44% reported income of between one and three minimum wages. The following occupations were reported: manager, engineer, cleaning services assistant, sales representative, housewife, housemaid, retired, seamstress, nurse, financial inspector, administrative assistant, hairdresser, plasterer, teacher and student (no category presented high frequency).

Six participants (24%) reported taking care of the patient every day. The other answers were: “three times a week”, “one weekend a month”, “during the week” (Table 2).

**Table 2:** Characteristics of volunteer caregivers.

Characteristics	Volunteer caregiver (N=21)
Time as a caregiver (months) (mean±SD)	27.7±41.9 (range: 3-180)
Weekly time of care (hours) (mean±SD)	46.8±57.8 (range: 2-168)
Zarit Burden Interview (mean±SD)	35.1±14.7 (range: 12-65)
Zarit Burden Interview "severity" - N (%) Up to moderate burden (£40) Moderate to severe burden	15 (71.4) 6 (28.6)
Help in activities of daily life (Yes) - N (%) Leisure Physical activity Finances Medication Personal hygiene Dress Feeding Other activities All activities	11 (48) 2 (9) 4 (17) 8 (35) 5 (22) 4 (17) 9 (39) 3 (13) 5 (22)
Support Networks (Yes) - N (%) Family Friends Neighbors Social group Paid caregiver Health professionals	13 (56) 4 (17) 1 (4) 6 (26) 9 (39) 4 (17)
How often uses the support networks - N (%) Daily or weekly Fortnightly, monthly or occasionally	12 (57) 9 (43)

Most caregivers (40%) stated they had been taking care of the patient for a period that ranged from three to 12 months. In terms of care profile, most participants (38%) indicated recruitment of daily assistance from other relatives or paid caregivers as additional help.

Twenty-one volunteer caregivers fully answered the ZBI. Fifteen (71.4%) caregivers presented up to moderate burden, and six (28.6%) reported moderate to severe burden (Table 2).

The correlation analysis between the ZBI and the other variables (i.e., age, number of children, schooling, time as a caregiver and weekly time of care) showed no significant results. However, a secondary correlation was found between time as caregiver and weekly time of care (*rho*=0.578, p=0.039).

## Discussion

The objectives of this study were to identify the sociodemographic profile of those individuals who attended the Support Group for Family Members of patients with Alzheimer’s disease at Hospital de Clínicas de Porto Alegre, and to verify the degree of burden generated from the care given to this kind of patient.

We observed that approximately half of the individuals who attended the group were non-caregivers and were seeking information about the disease. Almost half of these individuals lived alone, and 58% had above the average monthly income, i.e., four to seven minimum wages of monthly income. This group was composed of older participants which is encouraging because it represents a group of elderly individuals that were being informed on Alzheimer’s disease and other dementias. As previously established, the main risk factor for developing Alzheimer’s disease is age, i.e., as an individual ages the chance of developing the disease increases. Getting information on the first signs and symptoms of the disease could help not only in early diagnosis but also in developing coping strategies, organizing financial issues, and planning other aspects of life while individuals are still considered legally capable of decision making. Furthermore, early detection of the disease might allow for treatment of physical and psychiatric causes and aid the search for psychosocial support, and pharmacological symptomatic treatments.^24^

Of the 21 participants in the caregiver group, most were married, had higher educational attainment and other occupations besides being caregivers. These various simultaneously roles may act as factors increasing perceived burden. However, the typical profile previously reported depicts a caregiver who does not work and is able to be devoted to patient care.^25^ Since the objective of the present study was the description of the profile of caregivers, the distribution of characteristics was not controlled. However, the number of professional caregivers was very small precluding any meaningful statistical analysis.

Age was the only variable that differentiated one subgroup from the other. Overall, the caregivers were younger than the non-caregivers. Because non-caregivers were individuals searching for information on dementia, it is no surprise that elderly people most frequently took part in this type of activity. On the other hand, caregivers were expected to be younger than patients.

Women predominated in both groups, where this is often the case because women have the habit of seeking expert help.^26^ The higher frequency of women caregivers has previously been observed.^9,10,12,27,28^ It has also been demonstrated that women show higher levels of burden as caregivers.^29^

No correlation between education and the ZBI was found, suggesting little or no effect of education on the perception of burden. However, the range of education was narrow. Age also did not correlate with burden. We did however observe a correlation between time as caregiver and weekly time of care, i.e., the longer the person has been a caregiver the more weekly time of care was spent. As the disease progresses and patient independence diminishes there is an increased need of care. This could be an explanation for the observed correlation.

The present study also sought to describe the degree of burden of the caregivers using the Zarit Burden Interview. The mean score on the ZBI was 35 (range 12-65), with 71.4% of subjects presenting up to moderate, and 28.6% moderate to severe, levels of burden. The intensity of burden among caregivers who were highly educated from middle to high socioeconomic background and who had spent less than a year on average as caregiver was high, suggesting these factors were not protective against distress.

Limitations of the study were the small sample size, the lack of control of the time participants had frequented the support group, and absence of the patients’ clinical information. Additionally, assessing caregivers who already attended a support group might have influenced the results by showing a lower impact of burden.

Future studies are warranted in larger samples of caregivers in Brazil to enhance understanding of the characteristics and distress of this group of individuals.
